# Uterine Smooth Muscle Tumor of Undetermined Malignant Potential (STUMP): A Diagnostic Challenge

**DOI:** 10.7759/cureus.73115

**Published:** 2024-11-06

**Authors:** Sanghamitra Jena, Neetesh K Sinha, Minakshi Mishra, Vinita Singh, Amitabh Kumar Upadhyay

**Affiliations:** 1 Surgical Oncology, Tata Main Hospital, Jamshedpur, IND; 2 Pathology, Tata Main Hospital, Jamshedpur, IND; 3 Obstetrics and Gynecology, Tata Main Hospital, Jamshedpur, IND; 4 Medical Oncology, Tata Main Hospital, Jamshedpur, IND

**Keywords:** immunohistochemistry, polyp, smooth muscle tumor, stump, uterine

## Abstract

Smooth muscle tumors of the uterus can range from benign leiomyoma (LM) to malignant leiomyosarcoma (LMS) histologically. Between these two entities lies a gray zone of smooth muscle tumors of undetermined malignant potential, commonly known as STUMP. Their diagnosis has always been a challenge for the pathologists. Once diagnosed, clinicians face difficulties in managing STUMP because of the lack of discrete treatment guidelines.

Here, we report a case of a 52-year-old lady who presented in an emergency with severe menorrhagia. On examination, the patient was pale and there was a 4x3 cm size polyp protruding through the internal ostium. Hysteroscopy with endometrial biopsy and polypectomy were done. The polypectomy specimen was diagnosed as STUMP after a thorough histopathological examination and immunohistochemistry (IHC). The specimen was re-examined in two other reputed institutes, as there was a dilemma between the diagnosis of STUMP/leiomyosarcoma. The patient was treated with a radical hysterectomy, considering the upstaged diagnosis of leiomyosarcoma. The final histopathological diagnosis of the hysterectomy specimen was STUMP. The patient did not receive any adjuvant therapy. Presently, she is on follow-up every six months and has not developed any recurrence till now.

The aim of reporting this case is to enrich the existing literature on this rare diagnosis and highlight the fact that STUMP can also present as a uterine polyp. This is probably the first case of STUMP presenting as a uterine polyp.

## Introduction

In 1973, Langley first described a uterine smooth muscle tumor of undetermined malignant potential, named STUMP [1]. Then, in 2014, WHO defined STUMP as a group of smooth muscle tumors of the uterus, which did not fulfill the criteria for diagnosis of either leiomyoma (LM) or leiomyosarcoma (LMS) [2]. They present clinically with the signs and symptoms resembling leiomyoma. The diagnosis is mostly established postoperatively with the expertise of the pathologists and extensive immunohistochemistry (IHC) panel testing. Since these tumors do not have high malignant potential but can recur and metastasize lately, vigilant long-term follow-up is required in the management of these cases.

Herein, we report a case of STUMP presenting as a uterine polyp. Its diagnosis and management were challenging for both the pathologists and the clinicians, because of the paucity of data on this topic.

## Case presentation

A 52-year-old lady presented to the emergency of the Obstetrics and Gynecology department with chief complaints of severe menorrhagia and a mass coming out of her internal os. She was G2P2A0, all by normal vaginal delivery. She had a history of heavy menstrual bleeding with the passage of clots for the last three years. The patient was receiving Nor-ethisterone (5 mg) thrice daily, but the symptoms did not subside. The bleeding had increased in severity and was associated with vaginal discharge in the last two months.

On examination, the lady was of average body habitus and had gross pallor. The abdominal examination did not reveal any abnormality. The uterus was 12 weeks in size, anteverted with bilateral fornices free per vaginal examination. Per speculum examination showed a mass of 4x3 cm in size, protruding through the ostium.

**Figure 1:**
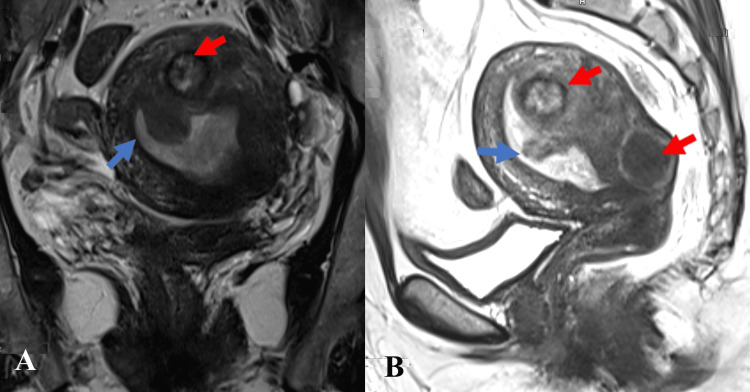
A) Coronal view; B) Sagittal view Coronal and sagittal T2W sequence of the pelvis showing a bulky uterus with T2 hypo-intense polypoid lesion (blue arrow) projecting into the endometrium arising from the submucosal portion of the posterior myometrium. There is a disruption of the posterior junctional zone at the base of the polypoidal lesion. Two T2 hypo-intense and hetero-intense intramural uterine fibroids (red arrow) were also noted.

The blood parameters were within normal limits except for hemoglobin, which was 6 g/dl. CA-125 and lactate dehydrogenase (LDH) were 40 units/ml and 210 units/l, respectively. The patient was transfused three units of whole blood and taken up for emergency hysteroscopy along with endometrial biopsy and excision of the uterine polyp. The surgery and postoperative period were uneventful. The histopathological diagnosis of the uterine polyp was STUMP. Since the diagnosis is rare, the slides and blocks were sent to two other institutes for review. In the meantime, imaging with contrast-enhanced computed tomography (CECT) of the chest and contrast-enhanced magnetic resonance imaging (MRI) of the abdomen and pelvis was done. CECT thorax showed no pulmonary metastases. MRI of the whole abdomen revealed a mildly enlarged uterus measuring 9x6.5x7.1 cm. An ill-defined altered signal intensity lesion was seen in the sub-mucosal location arising from the posterior myometrium, measuring around 2.4x1.4x2.1 cm (Figure 1). Disruption of the junctional zone was seen adjacent to the submucosal extension. Myometrial involvement was limited to 50% of myometrial thickness. Altered signal intensity of endometrial thickening measuring 4.7x1.2x4.1 cm was present. Three other leiomyomas were reported, one present in the upper body and two in the lower body of the uterus. The cervix and bilateral ovaries were normal. There was no ascites or significant pelvic or retroperitoneal lymphadenopathy.

Since there was confusion about the diagnosis between STUMP and leiomyosarcoma and the review of histological blocks sent outside was taking time, the patient was planned for a radical hysterectomy and bilateral salpingo-oophorectomy in the multidisciplinary tumor board. During the radical hysterectomy, bilateral pelvic lymph nodes seemed to be enlarged. So, they were sampled and sent for a frozen section. The frozen section report was negative for malignancy.

**Figure 2:**
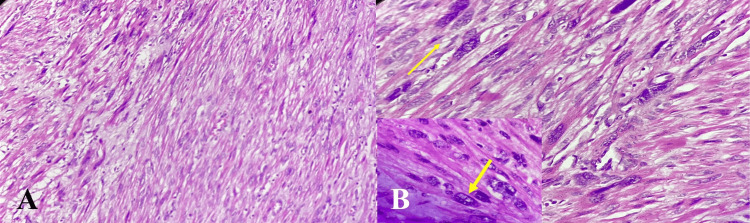
A) Histology from the solid mass lesion in the uterus showing a spindle cell cellular neoplasm (40X H&E); B) High power view showing cellular spindle cell neoplasm with moderate to marked nuclear atypia. A few scattered bizarre nuclei are also seen. No necrosis is seen. Mitotic count is 2/10 hpf (200X H&E). B): The thin yellow arrow showing cellular areas in the spindle cell neoplasm. The thick yellow area showing enlarged, pleomorphic nuclei with multinucleation.

The final histopathological examination showed spindle cell neoplasm in the submucosal tumor. The other three lesions were intramural fibroid. The spindle cell tumor had normal to significant cellular areas, with diffuse moderate to marked nuclear atypia, mostly with vesicular nuclei and prominent nucleoli. A few scattered bizarre nuclei and multinucleated cells were seen. Other areas showed minimal to mild nuclear atypia and a few apoptotic bodies. Coagulative tumor cell necrosis (CTCN) was not identified. There were no lymphovascular emboli. The mitotic index (MI) was 2-3/10 high power fields (HPFs). No significant demarcation with the normal myometrium was noted and the serosa was 1.5 cm from the tumor. The surface of the tumor showed areas of a fibrino-inflammatory exudate and fairly preserved normal endometrial glands (Figure 2).

**Figure 3:**
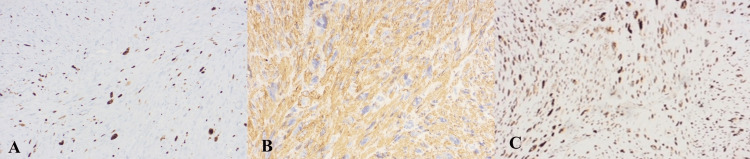
A) IHC Ki-67 X 200, immuno-reactive in 18-20 % of lesional cells; B) IHC SMA X 200, immuno-reactive, Score 4+ in lesional cells: C) IHC P53, 50-60% cells show positivity with moderate intensity (regarded as wild type staining) IHC: immunohistochemistry

The diagnosis of STUMP was supported by IHC, which was positive for H-Caldesmon and Desmin. Estrogen receptor (ER) was positive (60% of cells with moderate intensity). P16 was patchy positive and P53 was positive with moderate intensity in 50-60% of cells. Ki-67 was immunoreactive in 18-20% of lesional cells. SMA was immunoreactive with a score of 4+ in lesional cells (Figure 3).

The patient is kept under surveillance at an interval of six months with clinical examination and imaging of the chest and abdomen.

## Discussion

The diagnosis of STUMP is made based on three histologic features, i.e. cellular atypia, CTCN, and MI [2]. The various combinations of these parameters are illustrated in Table 1.

**Table 1:** Various combinations of histologic features for the diagnosis of STUMP Source: [2] STUMP: smooth muscle tumors of undetermined malignant potential

Cellular atypia	CTCN	MI
-	+	<10/10 HPFs
Moderate to severe	-	<10/10 HPFs
-	-	>15/10 HPFs

Gupta et al. added four other pathological features, such as vascular space invasion, permeative margins, depth of myometrial invasion, and the presence of epithelioid and myxoid histology, for the prognostication of STUMP [3].

IHC is mandatory for differentiating STUMP from LM and LMS [4-6]. Still, there may be an overlap of expression of IHC markers in the three groups as illustrated in Table 2.

**Table 2:** IHC expression in LM, STUMP, and LMS Sources: [4-6] IHC: immunohistochemistry; LM: leiomyoma; STUMP: smooth muscle tumors of undetermined malignant potential; LMS: malignant leiomyosarcoma

IHC	LM	STUMP	LMS
ER	++	++	-/+
PR	++	++	-/+
P16	-/+	-/+	++
P53	-/+	-/+	++

STUMP can present in any age group with symptoms of menorrhagia, abdominal mass, or pelvic pain [7]. However, its presentation as a uterine polyp protruding through the ostium has not yet been reported in the available literature. Our case is probably the first case with such a presentation.

It is difficult to differentiate STUMP from the other two entities clinically, and imaging may be of some help. Ultrasound findings are usually not very specific. MRI with irregular tumor margin suggests LMS and STUMP rather than LM. MRI grading systems based on T2-weighted imaging, diffusion-weighted imaging, and apparent diffusion coefficient values are also useful in diagnosis [8]. According to these parameters, benign and degenerated LM are grades I-III, and grades IV-V are variants of LM or STUMP. Position emission tomography (PET) is more specific compared to MRI. Maximal standardized uptake values and the metabolic tumor-to-necrosis ratio are significantly higher in LMS and STUMP than in LM. LMS and STUMP exhibit a characteristic “hollow ball” sign, corresponding to tumor necrosis in PET [9].

The standard treatment is surgery with a tumor-free margin. It can be myomectomy or hysterectomy with or without bilateral salpingo-oophorectomy per the patient’s consensus [10]. Myomectomy is usually done in patients willing for fertility preservation. However, they should be followed up closely as there may be chances of recurrence [11].

There is no proven role of adjuvant chemotherapy, radiotherapy, or hormonal therapy in patients treated adequately by surgery. Few studies have highlighted the use of chemotherapy and hormonal therapy, in cases of recurrence not salvageable by surgery and in distant metastases. The commonly used chemotherapeutic agents are doxorubicin, ifosfamide, cisplatin, gemcitabine, and docetaxel [12,13]. Progesterone receptor modulators, aromatase inhibitors, and gonadotropin-releasing hormone analogs are hormonal agents in receptor-positive STUMPs [14].

STUMP has an overall good prognosis compared to LMS, with five-year survival ranging from 60-90% [11]. But it can recur as early as 50 months to as late as 9 to 10 years [15]. The recurrence can be as STUMP or LMS. Given the extended interval between recurrences and the potential for treating recurrences with repeat surgery, these patients should undergo long-term follow-up for up to ten years. The surveillance is usually done with clinical examination and imaging every six months for five years and annually afterward [15,16].

## Conclusions

The incidence of STUMP is rare. The difficulty in its diagnosis demands pathologists’ expertise. As there is a lack of standard protocol for managing such cases, this case report will be an addition to the research done on this interesting topic. A multidisciplinary approach is helpful in managing such cases.
